# Equipping school counsellors to support students with eating disorders

**DOI:** 10.1186/2050-2974-1-S1-O63

**Published:** 2013-11-14

**Authors:** Colleen Alford, Joanne Titterton

**Affiliations:** 1 Eating Disorders Service, The Children's Hospital at Westmead, Australia

## 

School counsellors are often the first point of contact when people are concerned about a young person's eating. The Children's Hospital at Westmead Eating Disorders Program in collaboration with The Children's Hospital School has developed an eating disorder education program for school counsellors. The aim of this program is to equip school counsellors with general knowledge about eating disorders as well as an understanding of the evidence based treatment - family based treatment, and how to support students in the school setting. It has been rolled out across Western Sydney in Department of Education schools and will shortly be facilitated in Catholic Schools as well. The program runs for 90 minutes and is facilitated by a Clinical Nurse Consultant, a Social Worker and a teacher. The response from school counsellors has been very positive and has resulted in greater consistency in understanding of Eating Disorders, timely referrals, and clearer establishment of treatment roles. This presentation will outline the process of establishing this program as well as examining the content of the educational program, and presenting the results collated from school counsellor feedback.

This abstract was presented in the **Understanding and Treating Eating Pathology** stream of the 2013 ANZAED Conference.

